# Advertising and the New York University

**Published:** 1855-05

**Authors:** 


*Advertising and the New York University.*

We have recently received several advertisements, with a request that they should be inserted in the Journal. If we were willing to be governed by the principles which seem to govern the newspaper press generally, we should insert them all without the least hesitation. We have ever doubted the correctness of the principle, however, and shall entirely repudiate it, so far as our own journal is concerned.

We shall publish no advertisements *merely* because they can be paid for. According to our ideas of morality, a publisher who, for a stipulated price, inserts an advertisement in his paper or journal, becomes in part at least responsible for the influence it may exert on his readers. It is in vain that the publisher attempts to shield himself under the plea that the advertiser is alone responsible for the influence of his advertisement; that his paper is only the *passive* medium through which it comes before the public. For it requires only a very limited observation to show that a large class of readers indissolubly connect the character of the editor and publisher with everything they find in their columns. And the expression is not uncommon, that there must be some truth in such and such statements or advertisements, or Mr. A. and Mr. G. would not have published them. We were led to these remarks by an advertisement of the Medical Department of the New York University, sent us a few days since with a request that we would give it two insertions in our journal. It covers three pages of foolscap, and would make something more than two pages of closely printed matter. After stating the number of students in attendance during the last session, the number of graduates, and the names of the professors, the "Course of Instruction" is announced as follows:

"The course of lectures given will be on anatomy—general, descriptive, surgical, and pathological; principles and operations of surgery; materia medica and therapeutics; institutes and practice of medicine; obstetrics, the diseases of women and children, with clinical midwifery; chemistry and physiology; clinical surgery; clinical medicine; clinical lectures on the diseases of the genito-urinary organs; clinical lectures on the diseases of women and children; clinical lectures on physical diagnosis."

Here we have announced *eleven* distinct "Courses of Lectures," with nothing to qualify in the least the necessary inference that they are all actually given during the regular college term; and nothing to prevent the reader from believing that the CLIINCAL instruction so prominently spoken of actually consists of instruction at the bed-side of the sick in one or more of the hospitals in that great commercial metropolis; and yet nothing could be more erroneous than such inferences.

Again, about one-half of this advertisement is made up of an account of five *College Cliniques*, held each week in the College building. The first enumerated is in the following words, viz.: "1st. *An Obstetric Clinique for the diseases of women and children*, on every Monday, from 2½ to 4¼ o'clock P.M., by Prof. Bedford. Since the organization of this Clinique in Oct., 1850, there have been actually presented to the classes of the University between *eight and nine thousand* cases of the most interesting diseases incident to women and children."

Since Oct., 1850, there have been *five* College terms in the University. If we allow twenty weeks for each term, which will include both their regular term and their preliminary courses, we shall have 100 weeks for the five years, and an aggregate of 200 hours devoted to these cliniques, by Prof. Bedford. Now if we take the whole number of cases stated to be eight thousand five hundred, it will give 42½ patients for each clinique hour, or little more than one patient for every minute and a half. The reader must remember that the advertisement says all these cases have been "actually presented to the classes of the University." Only think of a professor of obstetrics "actually" presenting a case of the "most interesting diseases of women and children" every minute and a-half, for two hours in succession, and that, too, for every week for five successive College terms! We should have thought the statements in this advertisement had been made up exclusively for *Western* use, on the supposition that the Faculty of the New York University had taken it for granted that Western publishers were a little more *green* than the grass on our prairies, had we not found the same with many others in their recently published Announcement for 1855-6.

In the latter, after specially enumerating 769 cases of diseases of females "introduced to the class during the session just closing," it is added, "that each of the above cases was presented to the class, and fully examined, together with the necessary discussion of its causes, symptoms, diagnosis, and treatment."

The session then "just closing" was one simply of four months, or at longest eighteen weeks. A clinique of two hours every Monday would give the professor thirty-six hours during the session, or little more than one patient for every *three minutes*, without including any of the "almost every variety of infantile diseas[illegible]," which was presented at the same cliniques. To present to a class of students, fully examine, and discuss the causes, symptoms, diagnosis, and treatment of an important disease in from 1½ to 3 minutes on the average! Pooh! where is Barnum? What a slow coach the telegraph is? But, seriously, when will professors, as well as all others, learn that honesty is the best policy, and that the world, after all, readily distinguishes between a bag of gas and a pile of gold. If the Medical Faculty of the New York University, or any other Medical School in the Union, will send us a plain and truthful advertisement of their next college session. we will insert it in our journal with great pleasure, and for a reasonable consideration. But such bombast and transparent misrepresentations as we have been alluding to we will not publish either for love or money.

